# Supplementary material for: Resolving the full spectrum of human genome variation using Linked-Reads
Source: Genome Res. 2019 Apr;29(4):635–45. doi: 10.1101/gr.234443.118 (PMC6442396; doi:10.1101/gr.234443.118)
Supplement: Supplemental Material [file supp_gr.234443.118_Supplemental_longranger-2.2.zip › longranger-2.2.tar.bz2!/lr2.2-public/lib/stan/cmdstan/stan/doc/api/html/files.html]

Stan: File List


|  |  |
| --- | --- |
|  | Stan  2.5.0  probability, sampling & optimization |


- Main Page
- Related Pages
- Namespaces
- Classes
- Files

- File List
- File Members


All Classes Namespaces Files Functions Variables Typedefs Enumerations Enumerator Friends Macros Pages

File List

Here is a list of all files with brief descriptions:

[detail level 12345678]

|  |  |
| --- | --- |
| src |  |
| stan |  |
| agrad |  |
| fwd |  |
| functions |  |
| abs.hpp |  |
| acos.hpp |  |
| acosh.hpp |  |
| asin.hpp |  |
| asinh.hpp |  |
| atan.hpp |  |
| atan2.hpp |  |
| atanh.hpp |  |
| bessel\_first\_kind.hpp |  |
| bessel\_second\_kind.hpp |  |
| binary\_log\_loss.hpp |  |
| binomial\_coefficient\_log.hpp |  |
| cbrt.hpp |  |
| ceil.hpp |  |
| cos.hpp |  |
| cosh.hpp |  |
| digamma.hpp |  |
| erf.hpp |  |
| erfc.hpp |  |
| exp.hpp |  |
| exp2.hpp |  |
| expm1.hpp |  |
| fabs.hpp |  |
| falling\_factorial.hpp |  |
| fdim.hpp |  |
| floor.hpp |  |
| fma.hpp |  |
| fmax.hpp |  |
| fmin.hpp |  |
| fmod.hpp |  |
| gamma\_p.hpp |  |
| gamma\_q.hpp |  |
| hypot.hpp |  |
| inv.hpp |  |
| inv\_cloglog.hpp |  |
| inv\_logit.hpp |  |
| inv\_sqrt.hpp |  |
| inv\_square.hpp |  |
| is\_inf.hpp |  |
| is\_nan.hpp |  |
| lbeta.hpp |  |
| lgamma.hpp |  |
| lmgamma.hpp |  |
| log.hpp |  |
| log10.hpp |  |
| log1m.hpp |  |
| log1m\_exp.hpp |  |
| log1m\_inv\_logit.hpp |  |
| log1p.hpp |  |
| log1p\_exp.hpp |  |
| log2.hpp |  |
| log\_diff\_exp.hpp |  |
| log\_falling\_factorial.hpp |  |
| log\_inv\_logit.hpp |  |
| log\_rising\_factorial.hpp |  |
| log\_sum\_exp.hpp |  |
| logit.hpp |  |
| modified\_bessel\_first\_kind.hpp |  |
| modified\_bessel\_second\_kind.hpp |  |
| multiply\_log.hpp |  |
| owens\_t.hpp |  |
| Phi.hpp |  |
| pow.hpp |  |
| primitive\_value.hpp |  |
| rising\_factorial.hpp |  |
| round.hpp |  |
| sin.hpp |  |
| sinh.hpp |  |
| sqrt.hpp |  |
| square.hpp |  |
| tan.hpp |  |
| tanh.hpp |  |
| tgamma.hpp |  |
| trunc.hpp |  |
| value\_of.hpp |  |
| matrix |  |
| columns\_dot\_product.hpp |  |
| columns\_dot\_self.hpp |  |
| crossprod.hpp |  |
| determinant.hpp |  |
| divide.hpp |  |
| dot\_product.hpp |  |
| dot\_self.hpp |  |
| inverse.hpp |  |
| log\_determinant.hpp |  |
| log\_softmax.hpp |  |
| log\_sum\_exp.hpp |  |
| mdivide\_left.hpp |  |
| mdivide\_left\_ldlt.hpp |  |
| mdivide\_left\_tri\_low.hpp |  |
| mdivide\_right.hpp |  |
| mdivide\_right\_tri\_low.hpp |  |
| multiply.hpp |  |
| multiply\_lower\_tri\_self\_transpose.hpp |  |
| qr\_Q.hpp |  |
| qr\_R.hpp |  |
| rows\_dot\_product.hpp |  |
| rows\_dot\_self.hpp |  |
| softmax.hpp |  |
| sort.hpp |  |
| sum.hpp |  |
| tcrossprod.hpp |  |
| to\_fvar.hpp |  |
| trace\_gen\_quad\_form.hpp |  |
| trace\_quad\_form.hpp |  |
| typedefs.hpp |  |
| operators |  |
| operator\_addition.hpp |  |
| operator\_division.hpp |  |
| operator\_equal.hpp |  |
| operator\_greater\_than.hpp |  |
| operator\_greater\_than\_or\_equal.hpp |  |
| operator\_less\_than.hpp |  |
| operator\_less\_than\_or\_equal.hpp |  |
| operator\_multiplication.hpp |  |
| operator\_not\_equal.hpp |  |
| operator\_subtraction.hpp |  |
| operator\_unary\_minus.hpp |  |
| prob |  |
| univariate |  |
| continuous |  |
| normal.hpp |  |
| functions.hpp |  |
| fvar.hpp |  |
| matrix.hpp |  |
| numeric\_limits.hpp |  |
| operators.hpp |  |
| rev |  |
| error\_handling |  |
| matrix |  |
| check\_pos\_definite.hpp |  |
| matrix.hpp |  |
| functions |  |
| abs.hpp |  |
| acos.hpp |  |
| acosh.hpp |  |
| as\_bool.hpp |  |
| asin.hpp |  |
| asinh.hpp |  |
| atan.hpp |  |
| atan2.hpp |  |
| atanh.hpp |  |
| bessel\_first\_kind.hpp |  |
| bessel\_second\_kind.hpp |  |
| binary\_log\_loss.hpp |  |
| cbrt.hpp |  |
| ceil.hpp |  |
| cos.hpp |  |
| cosh.hpp |  |
| digamma.hpp |  |
| erf.hpp |  |
| erfc.hpp |  |
| exp.hpp |  |
| exp2.hpp |  |
| expm1.hpp |  |
| fabs.hpp |  |
| falling\_factorial.hpp |  |
| fdim.hpp |  |
| floor.hpp |  |
| fma.hpp |  |
| fmax.hpp |  |
| fmin.hpp |  |
| fmod.hpp |  |
| gamma\_p.hpp |  |
| gamma\_q.hpp |  |
| hypot.hpp |  |
| ibeta.hpp |  |
| if\_else.hpp |  |
| inv.hpp |  |
| inv\_cloglog.hpp |  |
| inv\_logit.hpp |  |
| inv\_sqrt.hpp |  |
| inv\_square.hpp |  |
| is\_inf.hpp |  |
| is\_nan.hpp |  |
| is\_uninitialized.hpp |  |
| lgamma.hpp |  |
| lmgamma.hpp |  |
| log.hpp |  |
| log10.hpp |  |
| log1m.hpp |  |
| log1m\_exp.hpp |  |
| log1p.hpp |  |
| log1p\_exp.hpp |  |
| log2.hpp |  |
| log\_diff\_exp.hpp |  |
| log\_falling\_factorial.hpp |  |
| log\_rising\_factorial.hpp |  |
| log\_sum\_exp.hpp |  |
| modified\_bessel\_first\_kind.hpp |  |
| modified\_bessel\_second\_kind.hpp |  |
| multiply\_log.hpp |  |
| owens\_t.hpp |  |
| Phi.hpp |  |
| Phi\_approx.hpp |  |
| pow.hpp |  |
| primitive\_value.hpp |  |
| rising\_factorial.hpp |  |
| round.hpp |  |
| sin.hpp |  |
| sinh.hpp |  |
| sqrt.hpp |  |
| square.hpp |  |
| step.hpp |  |
| tan.hpp |  |
| tanh.hpp |  |
| tgamma.hpp |  |
| trunc.hpp |  |
| value\_of.hpp |  |
| internal |  |
| ddv\_vari.hpp |  |
| dv\_vari.hpp |  |
| dvd\_vari.hpp |  |
| dvv\_vari.hpp |  |
| matrix\_vari.hpp |  |
| precomp\_v\_vari.hpp |  |
| precomputed\_gradients.hpp |  |
| v\_vari.hpp |  |
| vd\_vari.hpp |  |
| vdd\_vari.hpp |  |
| vdv\_vari.hpp |  |
| vector\_vari.hpp |  |
| vv\_vari.hpp |  |
| vvd\_vari.hpp |  |
| vvv\_vari.hpp |  |
| matrix |  |
| crossprod.hpp |  |
| determinant.hpp |  |
| divide.hpp |  |
| dot\_product.hpp |  |
| dot\_self.hpp |  |
| Eigen\_NumTraits.hpp |  |
| grad.hpp |  |
| initialize\_variable.hpp |  |
| LDLT\_alloc.hpp |  |
| LDLT\_factor.hpp |  |
| log\_determinant.hpp |  |
| log\_determinant\_ldlt.hpp |  |
| log\_determinant\_spd.hpp |  |
| log\_softmax.hpp |  |
| log\_sum\_exp.hpp |  |
| mdivide\_left.hpp |  |
| mdivide\_left\_ldlt.hpp |  |
| mdivide\_left\_spd.hpp |  |
| mdivide\_left\_tri.hpp |  |
| multiply.hpp |  |
| multiply\_lower\_tri\_self\_transpose.hpp |  |
| quad\_form.hpp |  |
| sd.hpp |  |
| softmax.hpp |  |
| sort.hpp |  |
| squared\_distance.hpp |  |
| stan\_print.hpp |  |
| stored\_gradient\_vari.hpp |  |
| sum.hpp |  |
| tcrossprod.hpp |  |
| to\_var.hpp |  |
| trace\_gen\_inv\_quad\_form\_ldlt.hpp |  |
| trace\_gen\_quad\_form.hpp |  |
| trace\_inv\_quad\_form\_ldlt.hpp |  |
| trace\_quad\_form.hpp |  |
| typedefs.hpp |  |
| value\_of.hpp |  |
| variance.hpp |  |
| ode |  |
| coupled\_ode\_system.hpp |  |
| operators |  |
| operator\_addition.hpp |  |
| operator\_divide\_equal.hpp |  |
| operator\_division.hpp |  |
| operator\_equal.hpp |  |
| operator\_greater\_than.hpp |  |
| operator\_greater\_than\_or\_equal.hpp |  |
| operator\_less\_than.hpp |  |
| operator\_less\_than\_or\_equal.hpp |  |
| operator\_minus\_equal.hpp |  |
| operator\_multiplication.hpp |  |
| operator\_multiply\_equal.hpp |  |
| operator\_not\_equal.hpp |  |
| operator\_plus\_equal.hpp |  |
| operator\_subtraction.hpp |  |
| operator\_unary\_decrement.hpp |  |
| operator\_unary\_increment.hpp |  |
| operator\_unary\_negative.hpp |  |
| operator\_unary\_not.hpp |  |
| operator\_unary\_plus.hpp |  |
| boost\_fpclassify.hpp |  |
| calculate\_chain.hpp |  |
| chainable.hpp |  |
| error\_handling.hpp |  |
| functions.hpp |  |
| internal.hpp |  |
| matrix.hpp |  |
| numeric\_limits.hpp |  |
| ode.hpp |  |
| operators.hpp |  |
| print\_stack.hpp |  |
| var.hpp |  |
| var\_stack.cpp |  |
| var\_stack.hpp |  |
| vari.hpp |  |
| autodiff.hpp |  |
| fwd.hpp |  |
| partials\_vari.hpp |  |
| rev.hpp |  |
| command |  |
| print.hpp |  |
| stanc\_helper.hpp |  |
| common |  |
| recorder |  |
| csv.hpp |  |
| filtered\_values.hpp |  |
| messages.hpp |  |
| no\_op.hpp |  |
| sum\_values.hpp |  |
| values.hpp |  |
| command.hpp |  |
| context\_factory.hpp |  |
| do\_bfgs\_optimize.hpp |  |
| do\_print.hpp |  |
| init\_adapt.hpp |  |
| init\_nuts.hpp |  |
| init\_static\_hmc.hpp |  |
| init\_windowed\_adapt.hpp |  |
| initialize\_state.hpp |  |
| print\_progress.hpp |  |
| recorder.hpp |  |
| run\_markov\_chain.hpp |  |
| sample.hpp |  |
| warmup.hpp |  |
| write\_error\_msg.hpp |  |
| write\_iteration.hpp |  |
| write\_iteration\_csv.hpp |  |
| write\_model.hpp |  |
| write\_stan.hpp |  |
| gm |  |
| arguments |  |
| arg\_adapt.hpp |  |
| arg\_adapt\_delta.hpp |  |
| arg\_adapt\_engaged.hpp |  |
| arg\_adapt\_gamma.hpp |  |
| arg\_adapt\_init\_buffer.hpp |  |
| arg\_adapt\_kappa.hpp |  |
| arg\_adapt\_t0.hpp |  |
| arg\_adapt\_term\_buffer.hpp |  |
| arg\_adapt\_window.hpp |  |
| arg\_bfgs.hpp |  |
| arg\_data.hpp |  |
| arg\_data\_file.hpp |  |
| arg\_dense\_e.hpp |  |
| arg\_diag\_e.hpp |  |
| arg\_diagnose.hpp |  |
| arg\_diagnostic\_file.hpp |  |
| arg\_engine.hpp |  |
| arg\_fail.hpp |  |
| arg\_fixed\_param.hpp |  |
| arg\_history\_size.hpp |  |
| arg\_hmc.hpp |  |
| arg\_id.hpp |  |
| arg\_init.hpp |  |
| arg\_init\_alpha.hpp |  |
| arg\_int\_time.hpp |  |
| arg\_iter.hpp |  |
| arg\_lbfgs.hpp |  |
| arg\_max\_depth.hpp |  |
| arg\_method.hpp |  |
| arg\_metric.hpp |  |
| arg\_newton.hpp |  |
| arg\_num\_samples.hpp |  |
| arg\_num\_warmup.hpp |  |
| arg\_nuts.hpp |  |
| arg\_optimize.hpp |  |
| arg\_optimize\_algo.hpp |  |
| arg\_output.hpp |  |
| arg\_output\_file.hpp |  |
| arg\_random.hpp |  |
| arg\_refresh.hpp |  |
| arg\_rwm.hpp |  |
| arg\_sample.hpp |  |
| arg\_sample\_algo.hpp |  |
| arg\_save\_iterations.hpp |  |
| arg\_save\_warmup.hpp |  |
| arg\_seed.hpp |  |
| arg\_static.hpp |  |
| arg\_stepsize.hpp |  |
| arg\_stepsize\_jitter.hpp |  |
| arg\_test.hpp |  |
| arg\_test\_grad\_eps.hpp |  |
| arg\_test\_grad\_err.hpp |  |
| arg\_test\_gradient.hpp |  |
| arg\_thin.hpp |  |
| arg\_tolerance.hpp |  |
| arg\_unit\_e.hpp |  |
| argument.hpp |  |
| argument\_parser.hpp |  |
| argument\_probe.hpp |  |
| categorical\_argument.hpp |  |
| list\_argument.hpp |  |
| singleton\_argument.hpp |  |
| unvalued\_argument.hpp |  |
| valued\_argument.hpp |  |
| grammars |  |
| bare\_type\_grammar.hpp |  |
| bare\_type\_grammar\_def.hpp |  |
| bare\_type\_grammar\_inst.cpp |  |
| common\_adaptors\_def.hpp |  |
| expression07\_grammar.hpp |  |
| expression07\_grammar\_def.hpp |  |
| expression07\_grammar\_inst.cpp |  |
| expression\_grammar.hpp |  |
| expression\_grammar\_def.hpp |  |
| expression\_grammar\_inst.cpp |  |
| functions\_grammar.hpp |  |
| functions\_grammar\_def.hpp |  |
| functions\_grammar\_inst.cpp |  |
| iterator\_typedefs.hpp |  |
| program\_grammar.hpp |  |
| program\_grammar\_def.hpp |  |
| program\_grammar\_inst.cpp |  |
| statement\_2\_grammar.hpp |  |
| statement\_2\_grammar\_def.hpp |  |
| statement\_2\_grammar\_inst.cpp |  |
| statement\_grammar.hpp |  |
| statement\_grammar\_def.hpp |  |
| statement\_grammar\_inst.cpp |  |
| term\_grammar.hpp |  |
| term\_grammar\_def.hpp |  |
| term\_grammar\_inst.cpp |  |
| var\_decls\_grammar.hpp |  |
| var\_decls\_grammar\_def.hpp |  |
| var\_decls\_grammar\_inst.cpp |  |
| whitespace\_grammar.hpp |  |
| whitespace\_grammar\_def.hpp |  |
| whitespace\_grammar\_inst.cpp |  |
| ast.hpp |  |
| ast\_def.cpp |  |
| compiler.hpp |  |
| error\_codes.hpp |  |
| generator.hpp |  |
| parser.hpp |  |
| io |  |
| json |  |
| json\_data.hpp |  |
| json\_data\_handler.hpp |  |
| json\_error.hpp |  |
| json\_handler.hpp |  |
| json\_parser.hpp |  |
| cmd\_line.hpp |  |
| csv\_writer.hpp |  |
| dump.hpp |  |
| json.hpp |  |
| mcmc\_writer.hpp |  |
| reader.hpp |  |
| stan\_csv\_reader.hpp |  |
| util.hpp |  |
| var\_context.hpp |  |
| writer.hpp |  |
| math |  |
| error\_handling |  |
| matrix |  |
| check\_cholesky\_factor.hpp |  |
| check\_cholesky\_factor\_corr.hpp |  |
| check\_column\_index.hpp |  |
| check\_corr\_matrix.hpp |  |
| check\_cov\_matrix.hpp |  |
| check\_ldlt\_factor.hpp |  |
| check\_lower\_triangular.hpp |  |
| check\_matching\_dims.hpp |  |
| check\_matching\_sizes.hpp |  |
| check\_multiplicable.hpp |  |
| check\_nonzero\_size.hpp |  |
| check\_ordered.hpp |  |
| check\_pos\_definite.hpp |  |
| check\_pos\_semidefinite.hpp |  |
| check\_positive\_ordered.hpp |  |
| check\_range.hpp |  |
| check\_row\_index.hpp |  |
| check\_simplex.hpp |  |
| check\_size\_match.hpp |  |
| check\_spsd\_matrix.hpp |  |
| check\_square.hpp |  |
| check\_std\_vector\_index.hpp |  |
| check\_symmetric.hpp |  |
| check\_unit\_vector.hpp |  |
| check\_vector.hpp |  |
| constraint\_tolerance.hpp |  |
| validate\_non\_negative\_index.hpp |  |
| check\_bounded.hpp |  |
| check\_consistent\_size.hpp |  |
| check\_consistent\_sizes.hpp |  |
| check\_equal.hpp |  |
| check\_finite.hpp |  |
| check\_greater.hpp |  |
| check\_greater\_or\_equal.hpp |  |
| check\_less.hpp |  |
| check\_less\_or\_equal.hpp |  |
| check\_nonnegative.hpp |  |
| check\_not\_nan.hpp |  |
| check\_positive.hpp |  |
| check\_positive\_finite.hpp |  |
| dom\_err.hpp |  |
| dom\_err\_vec.hpp |  |
| functions |  |
| abs.hpp |  |
| as\_bool.hpp |  |
| bessel\_first\_kind.hpp |  |
| bessel\_second\_kind.hpp |  |
| binary\_log\_loss.hpp |  |
| binomial\_coefficient\_log.hpp |  |
| digamma.hpp |  |
| dist.hpp |  |
| divide.hpp |  |
| dot.hpp |  |
| dot\_self.hpp |  |
| exp2.hpp |  |
| falling\_factorial.hpp |  |
| fdim.hpp |  |
| gamma\_p.hpp |  |
| gamma\_q.hpp |  |
| ibeta.hpp |  |
| if\_else.hpp |  |
| int\_step.hpp |  |
| inv.hpp |  |
| inv\_cloglog.hpp |  |
| inv\_logit.hpp |  |
| inv\_sqrt.hpp |  |
| inv\_square.hpp |  |
| inverse\_softmax.hpp |  |
| is\_inf.hpp |  |
| is\_nan.hpp |  |
| is\_uninitialized.hpp |  |
| lbeta.hpp |  |
| lgamma.hpp |  |
| lmgamma.hpp |  |
| log1m.hpp |  |
| log1m\_exp.hpp |  |
| log1m\_inv\_logit.hpp |  |
| log1p.hpp |  |
| log1p\_exp.hpp |  |
| log2.hpp |  |
| log\_diff\_exp.hpp |  |
| log\_falling\_factorial.hpp |  |
| log\_inv\_logit.hpp |  |
| log\_rising\_factorial.hpp |  |
| log\_sum\_exp.hpp |  |
| logical\_and.hpp |  |
| logical\_eq.hpp |  |
| logical\_gt.hpp |  |
| logical\_gte.hpp |  |
| logical\_lt.hpp |  |
| logical\_lte.hpp |  |
| logical\_negation.hpp |  |
| logical\_neq.hpp |  |
| logical\_or.hpp |  |
| logit.hpp |  |
| max.hpp |  |
| min.hpp |  |
| modified\_bessel\_first\_kind.hpp |  |
| modified\_bessel\_second\_kind.hpp |  |
| modulus.hpp |  |
| multiply\_log.hpp |  |
| owens\_t.hpp |  |
| Phi.hpp |  |
| Phi\_approx.hpp |  |
| primitive\_value.hpp |  |
| promote\_scalar.hpp |  |
| promote\_scalar\_type.hpp |  |
| rising\_factorial.hpp |  |
| scaled\_add.hpp |  |
| sign.hpp |  |
| square.hpp |  |
| step.hpp |  |
| sub.hpp |  |
| sum.hpp |  |
| trigamma.hpp |  |
| value\_of.hpp |  |
| matrix |  |
| meta |  |
| index\_type.hpp |  |
| value\_type.hpp |  |
| accumulator.hpp |  |
| add.hpp |  |
| append\_col.hpp |  |
| append\_row.hpp |  |
| array\_builder.hpp |  |
| assign.hpp |  |
| block.hpp |  |
| cholesky\_decompose.hpp |  |
| col.hpp |  |
| cols.hpp |  |
| columns\_dot\_product.hpp |  |
| columns\_dot\_self.hpp |  |
| common\_type.hpp |  |
| containers\_conversion.hpp |  |
| crossprod.hpp |  |
| cumulative\_sum.hpp |  |
| determinant.hpp |  |
| diag\_matrix.hpp |  |
| diag\_post\_multiply.hpp |  |
| diag\_pre\_multiply.hpp |  |
| diagonal.hpp |  |
| dims.hpp |  |
| distance.hpp |  |
| divide.hpp |  |
| dot\_product.hpp |  |
| dot\_self.hpp |  |
| Eigen.hpp |  |
| eigenvalues\_sym.hpp |  |
| eigenvectors\_sym.hpp |  |
| elt\_divide.hpp |  |
| elt\_multiply.hpp |  |
| exp.hpp |  |
| fill.hpp |  |
| get\_base1.hpp |  |
| get\_base1\_lhs.hpp |  |
| get\_lp.hpp |  |
| head.hpp |  |
| initialize.hpp |  |
| inverse.hpp |  |
| inverse\_spd.hpp |  |
| LDLT\_factor.hpp |  |
| log.hpp |  |
| log\_determinant.hpp |  |
| log\_determinant\_ldlt.hpp |  |
| log\_determinant\_spd.hpp |  |
| log\_softmax.hpp |  |
| log\_sum\_exp.hpp |  |
| max.hpp |  |
| mdivide\_left.hpp |  |
| mdivide\_left\_ldlt.hpp |  |
| mdivide\_left\_spd.hpp |  |
| mdivide\_left\_tri.hpp |  |
| mdivide\_left\_tri\_low.hpp |  |
| mdivide\_right.hpp |  |
| mdivide\_right\_ldlt.hpp |  |
| mdivide\_right\_spd.hpp |  |
| mdivide\_right\_tri.hpp |  |
| mdivide\_right\_tri\_low.hpp |  |
| mean.hpp |  |
| meta.hpp |  |
| min.hpp |  |
| minus.hpp |  |
| multiply.hpp |  |
| multiply\_lower\_tri\_self\_transpose.hpp |  |
| num\_elements.hpp |  |
| prod.hpp |  |
| promote\_common.hpp |  |
| promote\_scalar.hpp |  |
| promote\_scalar\_type.hpp |  |
| promoter.hpp |  |
| qr\_Q.hpp |  |
| qr\_R.hpp |  |
| quad\_form.hpp |  |
| quad\_form\_diag.hpp |  |
| rank.hpp |  |
| resize.hpp |  |
| row.hpp |  |
| rows.hpp |  |
| rows\_dot\_product.hpp |  |
| rows\_dot\_self.hpp |  |
| sd.hpp |  |
| segment.hpp |  |
| singular\_values.hpp |  |
| size.hpp |  |
| softmax.hpp |  |
| sort.hpp |  |
| sort\_indices.hpp |  |
| squared\_distance.hpp |  |
| stan\_print.hpp |  |
| sub\_col.hpp |  |
| sub\_row.hpp |  |
| subtract.hpp |  |
| sum.hpp |  |
| tail.hpp |  |
| tcrossprod.hpp |  |
| trace.hpp |  |
| trace\_gen\_inv\_quad\_form\_ldlt.hpp |  |
| trace\_gen\_quad\_form.hpp |  |
| trace\_inv\_quad\_form\_ldlt.hpp |  |
| trace\_quad\_form.hpp |  |
| transpose.hpp |  |
| typedefs.hpp |  |
| variance.hpp |  |
| meta |  |
| index\_type.hpp |  |
| value\_type.hpp |  |
| ode |  |
| coupled\_ode\_observer.hpp |  |
| coupled\_ode\_system.hpp |  |
| integrate\_ode.hpp |  |
| constants.hpp |  |
| error\_handling.hpp |  |
| functions.hpp |  |
| matrix.hpp |  |
| matrix\_error\_handling.hpp |  |
| meta.hpp |  |
| ode.hpp |  |
| rep\_array.hpp |  |
| rep\_matrix.hpp |  |
| rep\_row\_vector.hpp |  |
| rep\_vector.hpp |  |
| seq\_view.hpp |  |
| mcmc |  |
| hmc |  |
| hamiltonians |  |
| base\_hamiltonian.hpp |  |
| dense\_e\_metric.hpp |  |
| dense\_e\_point.hpp |  |
| diag\_e\_metric.hpp |  |
| diag\_e\_point.hpp |  |
| ps\_point.hpp |  |
| unit\_e\_metric.hpp |  |
| unit\_e\_point.hpp |  |
| integrators |  |
| base\_integrator.hpp |  |
| base\_leapfrog.hpp |  |
| expl\_leapfrog.hpp |  |
| nuts |  |
| adapt\_dense\_e\_nuts.hpp |  |
| adapt\_diag\_e\_nuts.hpp |  |
| adapt\_unit\_e\_nuts.hpp |  |
| base\_nuts.hpp |  |
| dense\_e\_nuts.hpp |  |
| diag\_e\_nuts.hpp |  |
| unit\_e\_nuts.hpp |  |
| static |  |
| adapt\_dense\_e\_static\_hmc.hpp |  |
| adapt\_diag\_e\_static\_hmc.hpp |  |
| adapt\_unit\_e\_static\_hmc.hpp |  |
| base\_static\_hmc.hpp |  |
| dense\_e\_static\_hmc.hpp |  |
| diag\_e\_static\_hmc.hpp |  |
| unit\_e\_static\_hmc.hpp |  |
| base\_hmc.hpp |  |
| base\_adaptation.hpp |  |
| base\_adapter.hpp |  |
| base\_mcmc.hpp |  |
| chains.hpp |  |
| covar\_adaptation.hpp |  |
| fixed\_param\_sampler.hpp |  |
| sample.hpp |  |
| stepsize\_adaptation.hpp |  |
| stepsize\_adapter.hpp |  |
| stepsize\_covar\_adapter.hpp |  |
| stepsize\_var\_adapter.hpp |  |
| var\_adaptation.hpp |  |
| windowed\_adaptation.hpp |  |
| memory |  |
| stack\_alloc.hpp |  |
| meta |  |
| likely.hpp |  |
| traits.hpp |  |
| model |  |
| model\_header.hpp |  |
| prob\_grad.hpp |  |
| util.hpp |  |
| optimization |  |
| bfgs.hpp |  |
| bfgs\_linesearch.hpp |  |
| bfgs\_update.hpp |  |
| lbfgs\_update.hpp |  |
| newton.hpp |  |
| prob |  |
| distributions |  |
| multivariate |  |
| continuous |  |
| dirichlet.hpp |  |
| gaussian\_dlm\_obs.hpp |  |
| inv\_wishart.hpp |  |
| lkj\_corr.hpp |  |
| lkj\_cov.hpp |  |
| matrix\_normal.hpp |  |
| multi\_gp.hpp |  |
| multi\_gp\_cholesky.hpp |  |
| multi\_normal.hpp |  |
| multi\_normal\_cholesky.hpp |  |
| multi\_normal\_prec.hpp |  |
| multi\_student\_t.hpp |  |
| wishart.hpp |  |
| discrete |  |
| categorical.hpp |  |
| categorical\_logit.hpp |  |
| multinomial.hpp |  |
| continuous.hpp |  |
| discrete.hpp |  |
| univariate |  |
| continuous |  |
| beta.hpp |  |
| cauchy.hpp |  |
| chi\_square.hpp |  |
| double\_exponential.hpp |  |
| exp\_mod\_normal.hpp |  |
| exponential.hpp |  |
| frechet.hpp |  |
| gamma.hpp |  |
| gumbel.hpp |  |
| inv\_chi\_square.hpp |  |
| inv\_gamma.hpp |  |
| logistic.hpp |  |
| lognormal.hpp |  |
| normal.hpp |  |
| pareto.hpp |  |
| pareto\_type\_2.hpp |  |
| rayleigh.hpp |  |
| scaled\_inv\_chi\_square.hpp |  |
| skew\_normal.hpp |  |
| student\_t.hpp |  |
| uniform.hpp |  |
| von\_mises.hpp |  |
| weibull.hpp |  |
| discrete |  |
| bernoulli.hpp |  |
| beta\_binomial.hpp |  |
| binomial.hpp |  |
| hypergeometric.hpp |  |
| neg\_binomial.hpp |  |
| neg\_binomial\_2.hpp |  |
| ordered\_logistic.hpp |  |
| poisson.hpp |  |
| continuous.hpp |  |
| discrete.hpp |  |
| multivariate.hpp |  |
| univariate.hpp |  |
| autocorrelation.hpp |  |
| autocovariance.hpp |  |
| constants.hpp |  |
| distributions.hpp |  |
| internal\_math.hpp |  |
| traits.hpp |  |
| transform.hpp |  |
| welford\_covar\_estimator.hpp |  |
| welford\_var\_estimator.hpp |  |
| agrad.hpp |  |
| common.hpp |  |
| gm.hpp |  |
| io.hpp |  |
| math.hpp |  |
| mcmc.hpp |  |
| memory.hpp |  |
| meta.hpp |  |
| model.hpp |  |
| prob.hpp |  |
| version.hpp |  |


---

[ Stan Home Page ]

*© 2011–2014,
Stan Development Team.*
